# Temporal transcriptomic changes in long non-coding RNAs and messenger RNAs involved in the host immune and metabolic response during *Toxoplasma gondii* lytic cycle

**DOI:** 10.1186/s13071-021-05140-3

**Published:** 2022-01-10

**Authors:** Sha-Sha Wang, Chun-Xue Zhou, Hany M. Elsheikha, Jun-Jun He, Feng-Cai Zou, Wen-Bin Zheng, Xing-Quan Zhu, Guang-Hui Zhao

**Affiliations:** 1 College of Veterinary Medicine, Northwest A&F University, Yangling, 712100 Shaanxi China; 2 State Key Laboratory of Veterinary Etiological Biology, Key Laboratory of Veterinary Parasitology of Gansu Province, Lanzhou Veterinary Research Institute, Chinese Academy of Agricultural Sciences, Lanzhou, 730046 Gansu China; 3 Department of Pathogen Biology, School of Basic Medical Sciences, Cheeloo College of Medicine, Shandong University, Jinan, 250100 Shandong China; 4 Faculty of Medicine and Health Sciences, School of Veterinary Medicine and Science, University of Nottingham, Loughborough, LE12 5RD UK; 5 Present Address: Key Laboratory of Veterinary Public Health of Higher Education of Yunnan Province, College of Veterinary Medicine, Yunnan Agricultural University, Kunming, 650201 Yunnan China; 6 College of Veterinary Medicine, Shanxi Agricultural University, Taigu, 030801 Shanxi China

**Keywords:** *Toxoplasma gondii*, RNA-seq, lncRNA, mRNA, Immune response

## Abstract

**Background:**

Long non-coding RNAs (lncRNAs) are important regulators of various biological and pathological processes, in particular the inflammatory response by modulating the transcriptional control of inflammatory genes. However, the role of lncRNAs in regulating the immune and inflammatory responses during infection with the protozoan parasite *Toxoplasma gondii* remains largely unknown.

**Methods:**

We performed a longitudinal RNA sequencing analysis of human foreskin fibroblast (HFF) cells infected by *T. gondii* to identify differentially expressed long non-coding RNAs (lncRNAs) and messenger RNAs (mRNAs), and dysregulated pathways over the course of *T. gondii* lytic cycle. The transcriptome data were validated by qRT-PCR.

**Results:**

RNA sequencing revealed significant transcriptional changes in the infected HFFs. A total of 697, 1234, 1499, 873, 1466, 561, 676 and 716 differentially expressed lncRNAs (DElncRNAs), and 636, 1266, 1843, 2303, 3022, 1757, 3088 and 2531 differentially expressed mRNAs (DEmRNAs) were identified at 1.5, 3, 6, 9, 12, 24, 36 and 48 h post-infection, respectively. Gene Ontology (GO) and Kyoto Encyclopedia of Genes and Genomes (KEGG) enrichment analysis of DElncRNAs and DEmRNAs revealed that *T. gondii* infection altered the expression of genes involved in the regulation of host immune response (e.g., cytokine–cytokine receptor interaction), receptor signaling (e.g., NOD-like receptor signaling pathway), disease (e.g., Alzheimer's disease), and metabolism (e.g., fatty acid degradation).

**Conclusions:**

These results provide novel information for further research on the role of lncRNAs in immune regulation of *T. gondii* infection.

**Graphical Abstract:**

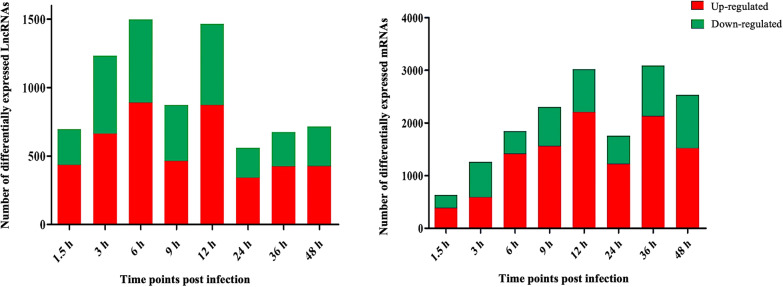

**Supplementary Information:**

The online version contains supplementary material available at 10.1186/s13071-021-05140-3.

## Background

*Toxoplasma gondii* is an obligate intracellular parasite which can infect many warm-blooded animals and is widespread in the human population [1, 2]. Human infection occurs mainly via ingestion of food or water contaminated with *T. gondii* oocysts or cysts, respectively [3]. Infected people with a compromised immune system can experience severe symptoms, such as headache, seizures or even death [4]. Unfortunately, there is no effective human vaccine to prevent infection, and drugs used to treat infected individuals have limitations [1, 2].

*Toxoplasma gondii* divides by endodyogeny inside an intracellular parasitophorous vacuole (PV) [5]. When the number of tachyzoites within the PV reaches a certain level, the tachyzoites egress from infected cells and infect new host cells in a process known as the lytic cycle [6]. Following host cell invasion, the proliferating tachyzoites of *T. gondii* establish an acute infection. In response to the pressure conferred by the host immune response, tachyzoites differentiate to slowly replicate bradyzoites and establish a lifelong latent infection, which can be reactivated in immunodeficient hosts [7]. The interaction of *T. gondii* with the host cells occurs at the transcriptional and post-transcriptional levels, which is fundamental for establishing a lytic or latent infection, but the molecular mechanisms that mediate these interactions remain poorly understood.

Several approaches, including transcriptomics [8–10], proteomics [11–13], and metabolomics [14], have been used to reveal the mechanisms that mediate the interaction between *T. gondii* and the host cell. Long non-coding RNAs (lncRNAs) have recently received increased attention because they play vital roles in cell development [15], chromatin modification [16], and immune regulation [17]. Changes in lncRNA expression have been associated with developmental defects [18], tumorigenesis [19], and autoimmune diseases [20]. There are a few studies on the expression of lncRNAs and their role in infectious diseases, such as those caused by viruses [21, 22]. Interestingly, deregulation of the expression of 996 lncRNAs and 109 messenger RNAs (mRNAs) has been demonstrated in human foreskin fibroblast (HFF) cells infected by *T. gondii* by using microarray analysis [23].

In the present study, we used RNA sequencing to obtain greater insight into the temporal changes in the expression of lncRNAs and mRNAs during the lytic cycle of *T. gondii* infection in HFF cells.

## Methods

### Parasites and cell culture

The HFF cells, purchased from the American Type Culture Collection (ATCC), were cultured in Dulbecco’s modified Eagle medium (DMEM) supplemented with 15% (vol/vol) fetal bovine serum (FBS), 100 U/ml penicillin, and 100 µg/ml streptomycin. The cell culture was maintained at 37 °C and 5% CO_2_. The RH (Type I) strain used in the present study was maintained in HFF cells as described previously [24].

### Sample collection, RNA extraction, and RNA sequencing (RNA-seq)

The HFF cells were infected with 10 × 10^6^ freshly egressed tachyzoites using a multiplicity of infection (MOI) of 1 (1 tachyzoite to 1 HFF cell). After 1.5 h, the culture medium was removed and replaced with fresh DMEM. The cultured cells were further incubated at 37 °C with 5% CO_2_. Then, infected cell samples were collected at 1.5, 3, 6, 9, 12, 24, 36 and 48 h post-infection (hpi) with *T. gondii*. Mock-infected cells were collected at 0 h. The experiment was carried out three times. The collected cell samples were stored at −80 °C until use for RNA extraction. Total RNA was extracted using TRIzol (Invitrogen, Carlsbad, CA, USA) according to the manufacturer’s instructions. The quantity and quality of the extracted RNA were determined by using the NanoDrop spectrophotometer and Agilent 2100 Bioanalyzer (Thermo Fisher Scientific, MA, USA), respectively. Samples with an RNA integrity number (RIN) ≥ 8.0 were used for RNA sequencing. The construction of sequencing libraries and sequencing were performed at Beijing Genomics Institute in Shenzhen using the BGISE500 platform.

### Data processing and differential expression analysis

The raw sequencing data were analyzed using SOAPnuke (v1.5.2) [25] to obtain high-quality reads. HISAT2 was then used to map the clean reads to the reference genome [26], followed by the application of Bowtie 2 (V2.2.5) [27] to align the clean reads to the gene set. A novel, coding and non-coding transcript database built by BGI (Beijing Genomic Institute in Shenzhen). RSEM (v1.2.12) was used to calculate gene expression levels [28], and differential expression analysis was performed using DESeq2 (v1.4.5) with *Q* value ≤ 0.05 [29].

### Function prediction of the differentially expressed lnRNAs

Gene Ontology (GO) (http://www.geneontology.org/) and the Kyoto Encyclopedia of Genes and Genomes (KEGG) (http://www.genome.jp/kegg/) enrichment analysis of the annotated host genes corresponding to differentially expressed lnRNAs (DElncRNAs) and DEmRNAs were performed by Phyper (https://en.wikipedia.org/wike/Hypergeometric_distribution) based on the hypergeometric test. The significance of the enriched GO terms and KEGG pathways was corrected by *Q* value with a threshold (*Q* value ≤ 0.05) using the Bonferroni method [30].

### Quantitative real-time PCR analysis

Validation of the differential expression of lnRNAs and mRNAs identified by RNA-seq was performed by using quantitative real-time PCR (qRT-PCR) analysis of eight randomly selected RNA samples. qRT-PCR was performed using the SYBR assay according to the manufacturer’s instructions in a total of 20 µl reaction volume, containing 1 µl of each primer, 2 µl cDNA, 6 µl nuclease-free H_2_O, and 10 µl master mix (Takara, Japan). Three replicates were performed for each gene, and the glyceraldehyde-3-phosphate dehydrogenase (*GAPDH*) gene was used as an internal control. The level of gene expression was performed by using the 2^−∆∆CT^ method. The primers used in the study (Table 1) were designed according to the sequences available in the NCBI database and synthesized at Sangon Biotech (Shanghai, China).

**Table 1:** The qRT-PCR primers used in the study

Target gene	Forward primer (5′–3′)	Reverse primer (5′–3′)
*IL-11*	CAGCGGACAGGGAAGGGTTAAAG	AGGCTCAGCACGACCAGGAC
*IL-32*	TGTGCTTCCCGAAGGTCCTCTC	TCTGCCAGGCTCGACATCACC
*CCL2*	AGGAACCGAGAGGCTGAGACTAAC	GGGAATGAAGGTGGCTGCTATGAG
*HOXA10*	TCCCACACTCGCCATCTCCTG	AACCAGCACCAAGCAAACACAAAG
*Loc101927226*	CATTACCTGCGTCACCTCCACAAG	TGTCACTGCTCCTCATCCTCCTG
*Loc107985080*	GAGAAGCAGGGCAGGAATGTGAC	CAGGCAGAACCGAAGGAAGGC
*LUCAT1*	GCAGCACTCAACTTGTATTCACTCAC	TGTTCACCACTGTACCCTCTACCC
*LINC00941*	CTAGGAGAGGGAGGGCAGAAGAAAG	TTGCTGTGAGCCAGGACCATATTAAG
*GAPDH*^a^	CACCACACCTTCTACAAC	TCTGGGTCATCTTCTCAC

^a^The *GAPDH* gene was used for mRNA and lncRNA normalization

### Statistical analysis

All statistical analyses were performed using GraphPad Prism 7 software (GraphPad Software, Inc., San Diego, CA, USA) and a value of *P* < 0.05 was considered statistically significant.

## Results

### Identification of DElncRNAs and DEmRNAs

**Fig. 1:**
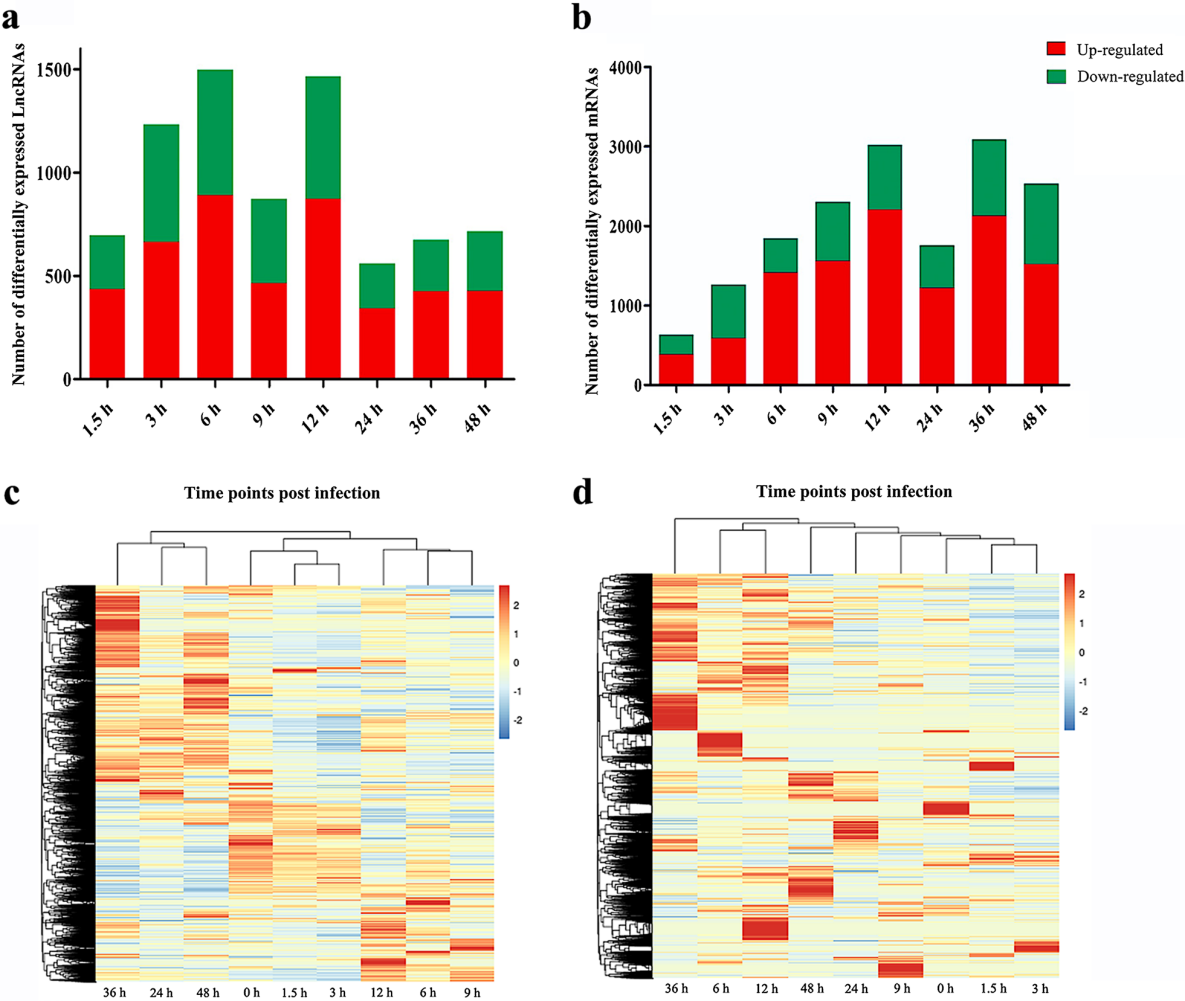
Differentially expressed (DE) lncRNAs (**a**) and mRNAs (**b**), and hierarchical clustering heatmaps of the DElncRNAs (**c**) and DEmRNAs (**d**) detected in HFF cells at different time points after *T. gondii* infection. The *x*-axis shows the time points after *T. gondii* infection and the *y*-axis shows the number of DElncRNAs and DEmRNAs. Red and green represent the upregulated and downregulated DElncRNAs and DEmRNAs, respectively

**Fig. 2:**
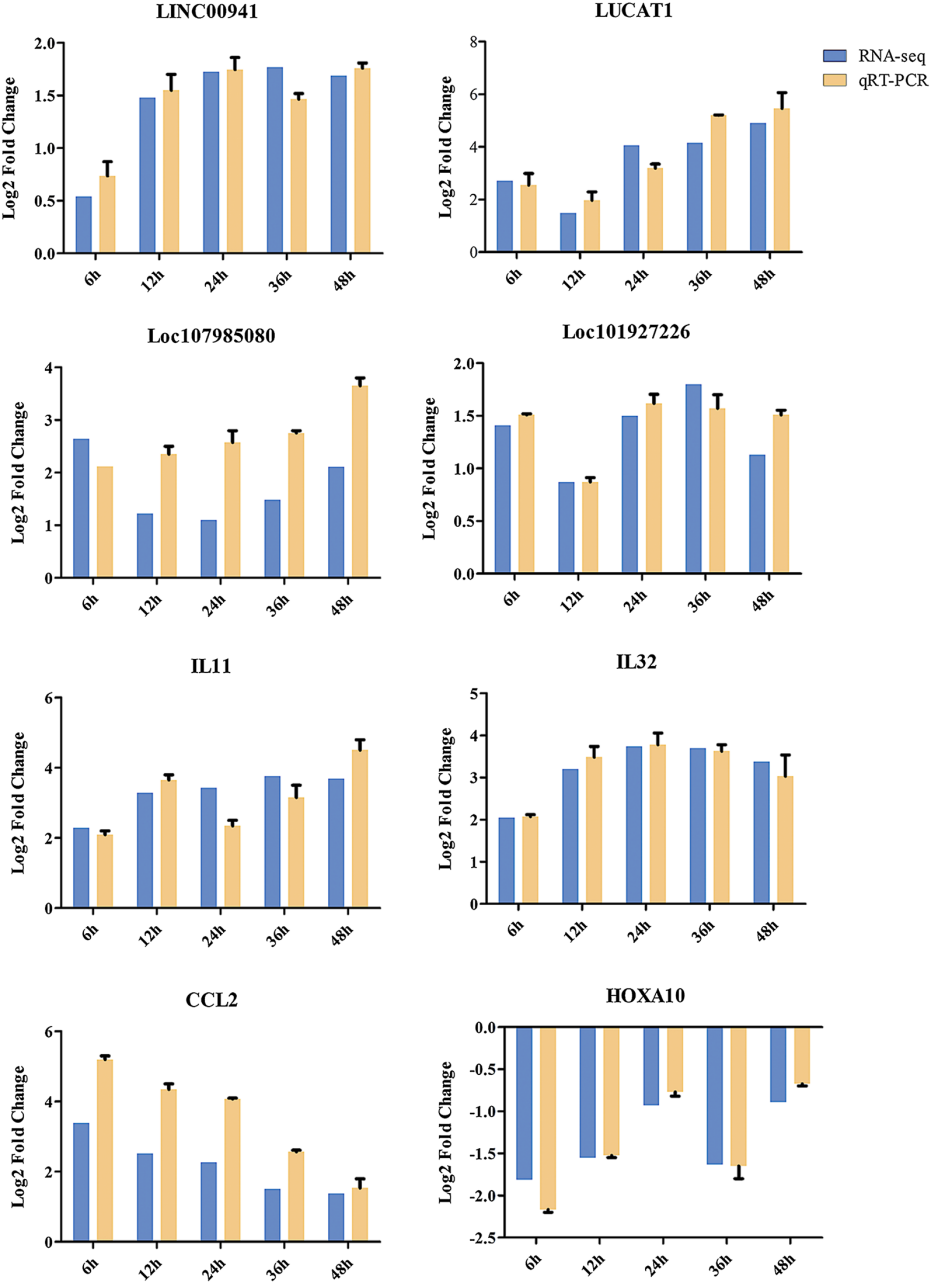
qRT-PCR-based validation of the expression of representative DElncRNAs (*LINC00941*, *LUCAT1*, *Loc107985080* and *Loc101927226*) and DEmRNAs (*IL-11*, *IL-32*, *CCL2* and *HOXA107*). The *x*-axis shows the names of the selected RNAs and the time points after infection. The *y*-axis shows the relative expression levels. The *GADPH* gene was used for mRNA and lncRNA normalization. qRT–PCR data are mean of three replicates ± standard deviation. Results obtained by RNA-seq analysis and qRT–PCR produced similar gene expression patterns

To characterize the dynamic expression and function of lncRNA and mRNAs in the lytic cycle of *T. gondii*, HFFs were collected for transcriptome analysis at 0, 1.5, 3, 6, 9, 12, 24, 36 and 48 hpi. *Q *value < 0.05 and |log_2_ fold change|> 1 were used as thresholds to identify differentially expressed genes (DEGs). The number and hierarchical clustering of DElncRNAs (Fig. 1a, c) and DEmRNAs (Fig. 1b, d) in infected cells at different time points compared with the sample at 0 h (control) are shown in Fig. 1. The DE-lncRNAs were clustered into three distinct groups as shown in the dendrogram on the top of the heatmap (Fig. 1c), indicating differences in the transcriptional profiles between the early (0 h, 1.5 h, and 3 h), middle (6 h, 9 h, and 12 h) and late (24 h, 36 h, and 48 h) stages of infection. Principal component analysis (PCA) of all identified lncRNAs (Additional file 1: Fig. S1a) and mRNAs (Additional file 1: Fig. S1b) further verified the reproducibility of these results. The volcano plots of DElncRNAs (Additional file 2: Fig. S2) and DEmRNAs (Additional file 3: Fig. S3) in infected cells at different time points compared with the sample at 0 h (control) are shown in Additional file 2: Fig. S2 and Additional file 3: Fig. S3. Our analysis identified 7,722 differently expressed lncRNAs (DElncRNAs), of which 697, 1,234, 1,499, 873, 1,466, 561, 676, and 716 lncRNAs were differently expressed at 1.5, 3, 6, 9, 12, 24, 36 and 48 hpi, respectively (Additional file 4: Table S1). A total of 16,446 differently expressed mRNAs (DEmRNAs) were identified; of which 636, 1,266, 1,843, 2,303, 3,022, 1,757, 3,088, and 2,531 were differentially expressed at 1.5, 3, 6, 9, 12, 24, 36 and 48 hpi, respectively (Additional file 5: Table S2). In addition, a total of 262 DElncRNAs (Additional file 6: Table S3) and 554 DEmRNAs (Additional file 7: Table S4) were co-expressed at all time points. The results of the RNA-seq were validated by analyzing the expression of four mRNAs (*IL-11*, *IL-32*, *CCL2*, and *HOXA10*) and four lncRNAs (*LINC00941*, *LUCAT1*, *Loc107985080*, and *Loc101927226*) at five time points (6, 12, 24, 36 and 48 hpi) using qRT-PCR. The qRT-PCR results showed that the expression profiles obtained by qRT-PCR analysis are consistent with the results of RNA-seq (Fig. 2), demonstrating the correctness of the RNA-seq data.

### The gene targets of DElncRNAs

**Fig. 3:**
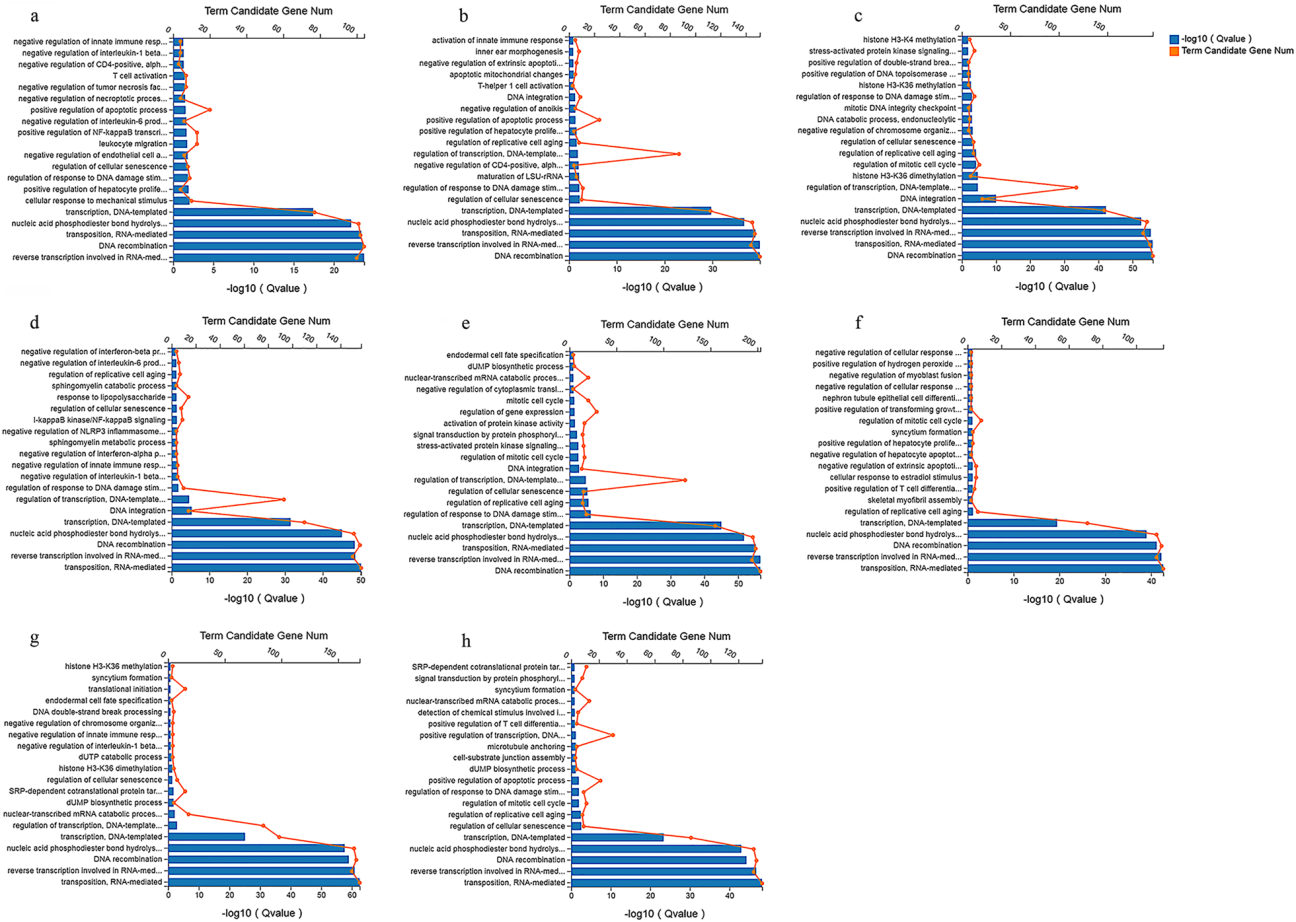
GO enrichment analysis of the target genes of the DElncRNAs in HFF cells at different time points after *T. gondii* infection. **a**–**h** The top 20 most-enriched GO terms in the biological process at 1.5, 3, 6, 9, 12, 24, 36 and 48 hpi, respectively. The *y*-axis represents the significantly enriched GO terms and the *x*-axis denotes the pathway enrichment and the number of DEmRNAs

**Fig. 4:**
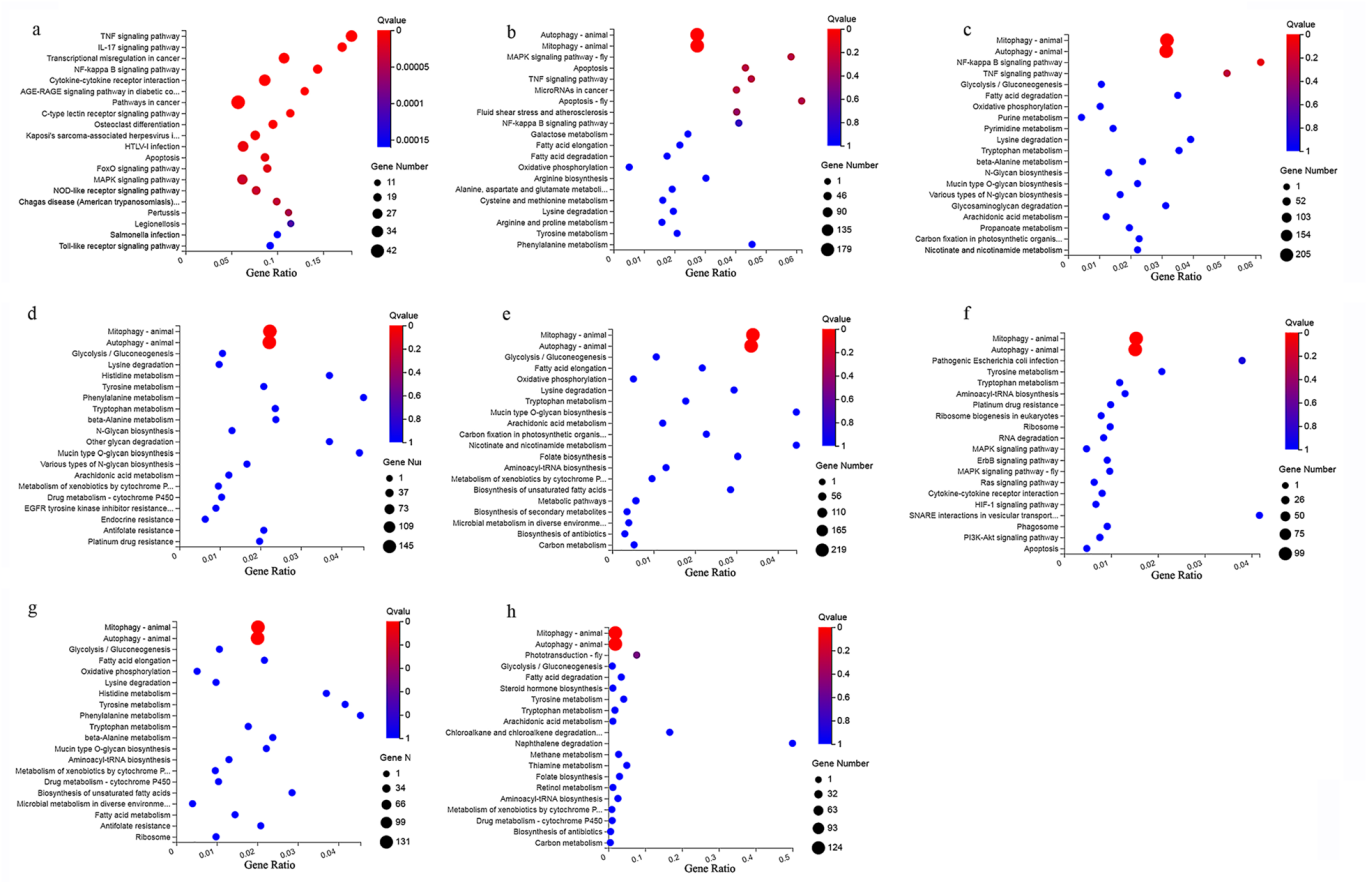
KEGG pathway analysis of the target genes of the DElncRNAs in HFF cells at different time points after *T. gondii* infection. **a**–**h** Scatterplots show the top 20 pathways at 1.5, 3, 6, 9, 12, 24, 36 and 48 hpi, respectively. The *x*-axis denotes the pathway enrichment. The *y*-axis represents the names of the significantly enriched pathways. The *P*-values are indicated by variations from blue to red, with darker blue denoting more significant difference

To identify the biological functions of DElncRNAs, GO enrichment analysis was performed using the gene target of DElncRNAs. The top 20 most-enriched GO terms in the biological processes are shown in Fig. 3. The biological processes of DElncRNAs at different groups were mainly involved in DNA recombination (GO:0006310), reverse transcription involved in RNA-mediated transposition (GO:0032199), transcription, DNA-templated (GO:0006351), and nucleic acid phosphodiester bond hydrolysis (GO:0090305) (Fig. 3). The top 20 most-enriched KEGG pathways of DElncRNAs are shown in Fig. 4. Most of the DElncRNAs, including *CLMAT3*, *TARID*, and *CARMN*, were involved in autophagy and mitophagy at different times. In addition, DElncRNAs were involved in immune-related pathways, such as the TNF signaling pathway (e.g., *CFLAR-AS1*, *CEBPB-AS1*, and *LOC100130476*), and the nuclear factor kappa B (NF-κB) signaling pathway (e.g., *C10orf55*, *CFLAR-AS1*, and *LOC646626*), and were significantly enriched at 1.5, 3 and 6 hpi. However, the metabolism-related pathways, such as fatty acid degradation (e.g., *CHKB-DT*) and tryptophan metabolism (e.g., *TMEM161B-AS1*, *LOC101929352* and *LOC105376957*) were enriched at other time points after infection. The upregulated and downregulated lncRNAs were mainly involved in autophagy and mitophagy (Additional file 8: Fig. S4 and Additional file 9: Fig. S5).

### Functional analysis of the DEmRNAs

**Fig. 5:**
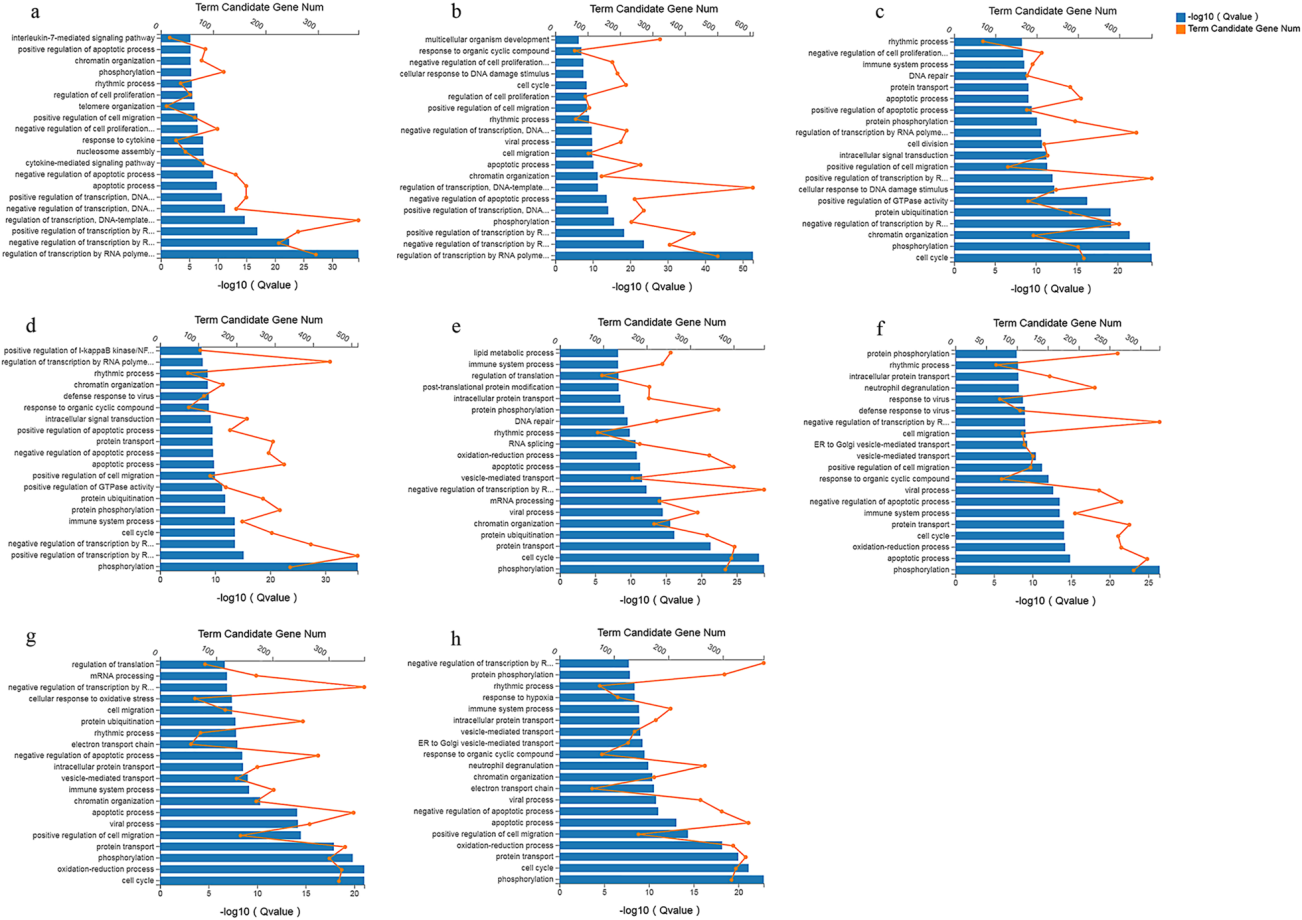
GO enrichment analysis of the DEmRNAs in HFF cells at different time points after *T. gondii* infection. **a**–**h** Scatterplots show the top 20 most-enriched GO terms in the biological process at 1.5, 3, 6, 9, 12, 24, 36 and 48 hpi, respectively. The *y*-axis represents the significantly enriched GO terms and the *x*-axis denotes the pathway enrichment and the number of DEmRNAs

**Fig. 6:**
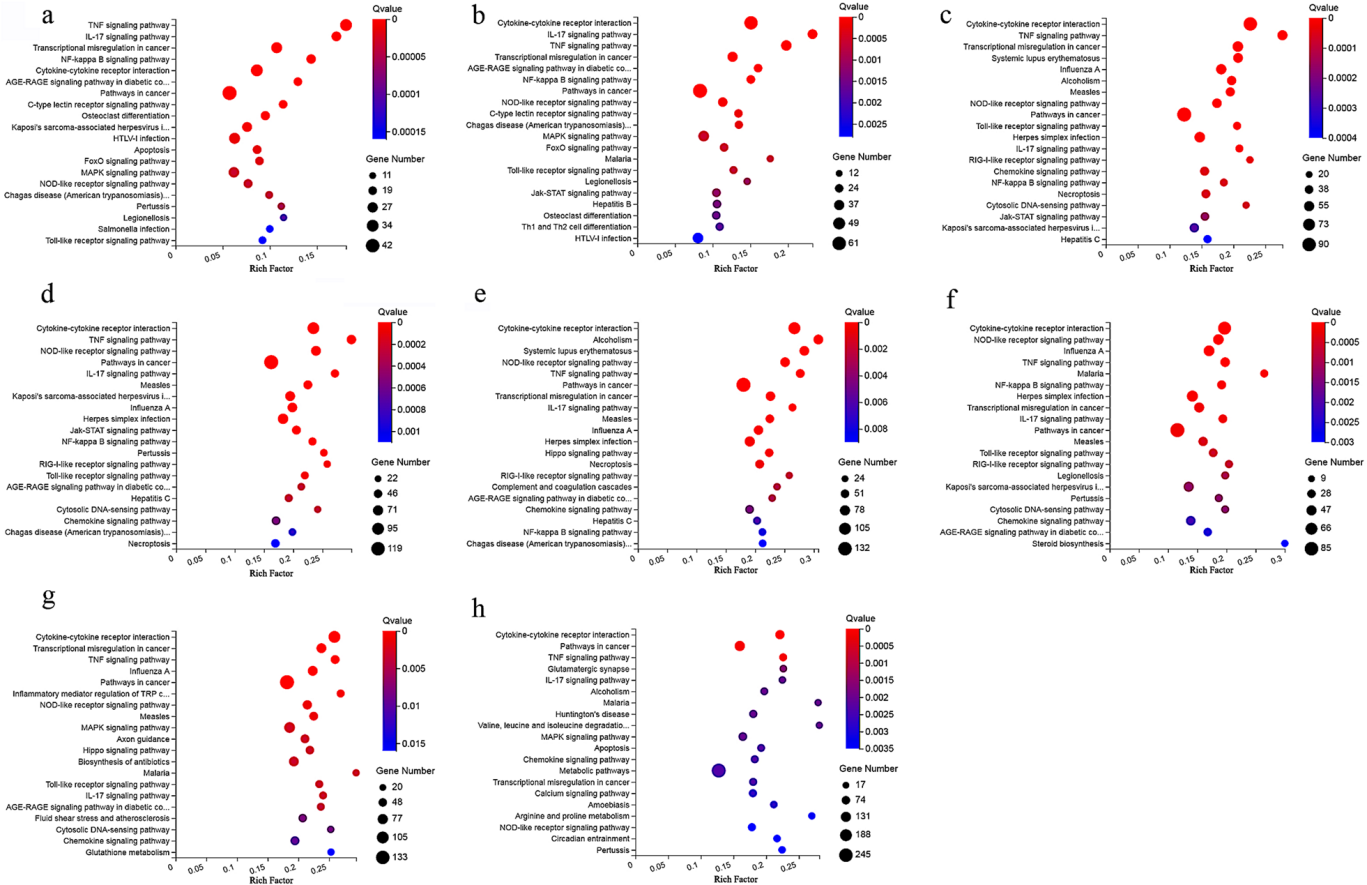
KEGG pathway analysis of the DEmRNAs in HFF cells at different time points after *T. gondii* infection. **a**–**h** Scatterplots show the top 20 pathways at 1.5, 3, 6, 9, 12, 24, 36 and 48 hpi, respectively. The *x*-axis denotes the pathway enrichment. The *y*-axis represents the names of the significantly enriched pathways. The *P*-values are indicated by variations from blue to red, with darker blue denoting more significant difference

The top 20 most-enriched biological processes are shown in Fig. 5. The biological processes of DEmRNAs at different time points were mainly involved in immune- or inflammation-related GO terms, such as the immune response (GO:0002376), inflammatory response (GO:0006954), defense response to virus (GO:0051607) and cytokine-mediated signaling pathway (GO:0019221). The top 20 most-enriched pathways of DEmRNAs are shown in Fig. 6. Most of the DEmRNAs were involved in immune-related pathways, such as cytokine-cytokine receptor interaction (e.g., *IL6*, *CXCL10* and *CSF2*), IL-17 signaling pathway (e.g., *CCL2*, *NFKB1* and *FOSL1*), TNF signaling pathway (e.g., *MAP3K8*, *IRF1* and *ICAM1*), and NOD-like receptor signaling pathway (e.g., *GBP1*, *GBP2* and *IFNAR2*). The upregulated mRNAs were mainly involved in immune-related pathways (Additional file 10: Fig. S6), and the downregulated mRNAs were mainly involved in metabolism-related pathways and neurodegenerative diseases-related pathways, such as Alzheimer's disease and Huntington's disease (Additional file 11: Fig. S7).

### Functional analysis of the gene targets of the co-expressed DElncRNAs

**Fig. 7:**
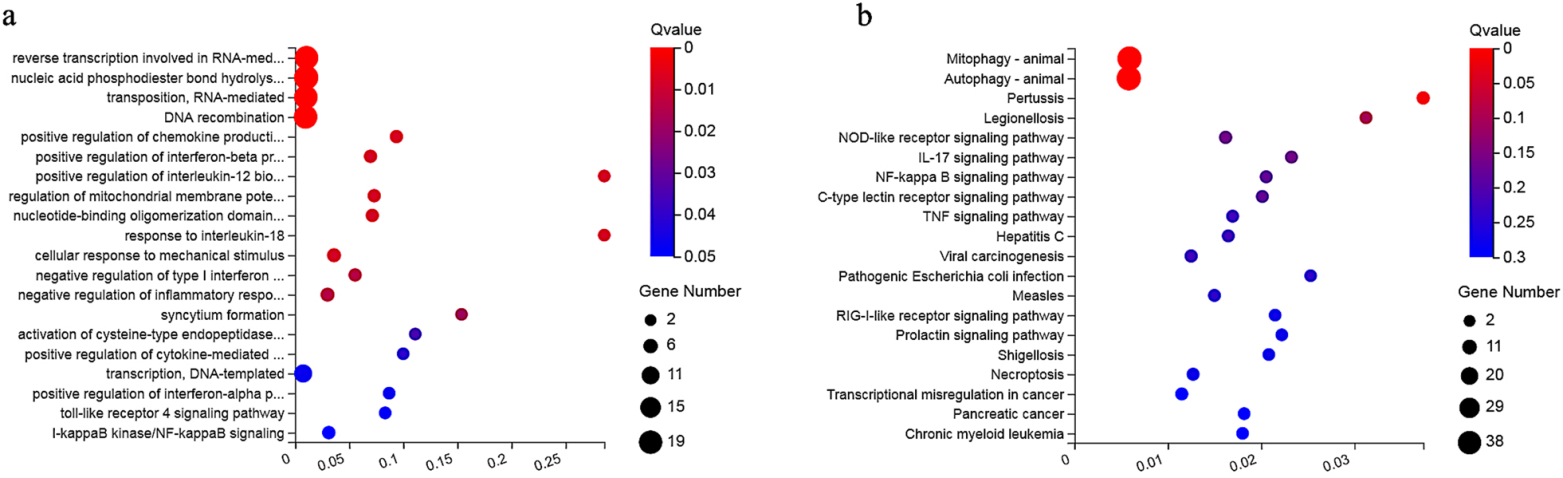
GO enrichment and KEGG pathway analyses of the target genes of co-expressed DElncRNAs in HFF cells after *T. gondii* infection. Scatterplots show **a** the 20 most-enriched GO terms in the biological process of target genes of DElncRNAs and **b** the top 20 pathways of the target genes of DElncRNAs. The *x*-axis denotes the pathway enrichment. The *y*-axis represents the names of the significantly enriched GO terms and pathways. The *P*-values are indicated by variations from blue to red, with darker blue denoting more significant difference

GO analysis and KEGG pathway enrichment were performed at different time points to identify the function of host genes corresponding to the DElncRNAs. The top 20 most-enriched biological processes are shown in Fig. 7a. They were mainly involved in reverse transcription linked to RNA-mediated transposition (e.g., *EGOT*, *LYRM4-AS1* and *SLCO4A1-AS1*), nucleic acid phosphodiester bond hydrolysis (e.g., *LYRM4-AS1*, *MSC-AS1* and *GNG12-AS1*), and DNA recombination (e.g., *PLCE1-AS1*, *ZNF667-AS1* and *LYRM4-AS1*). The top 20 most-enriched pathways of DElncRNAs are shown in Fig. 7b. Most of DElncRNAs were involved in autophagy and mitophagy (e.g., *LINC00173*, *LUCAT1* and *ITGB1-DT*).

### Functional analysis of the co-expressed DEmRNAs

**Fig. 8:**
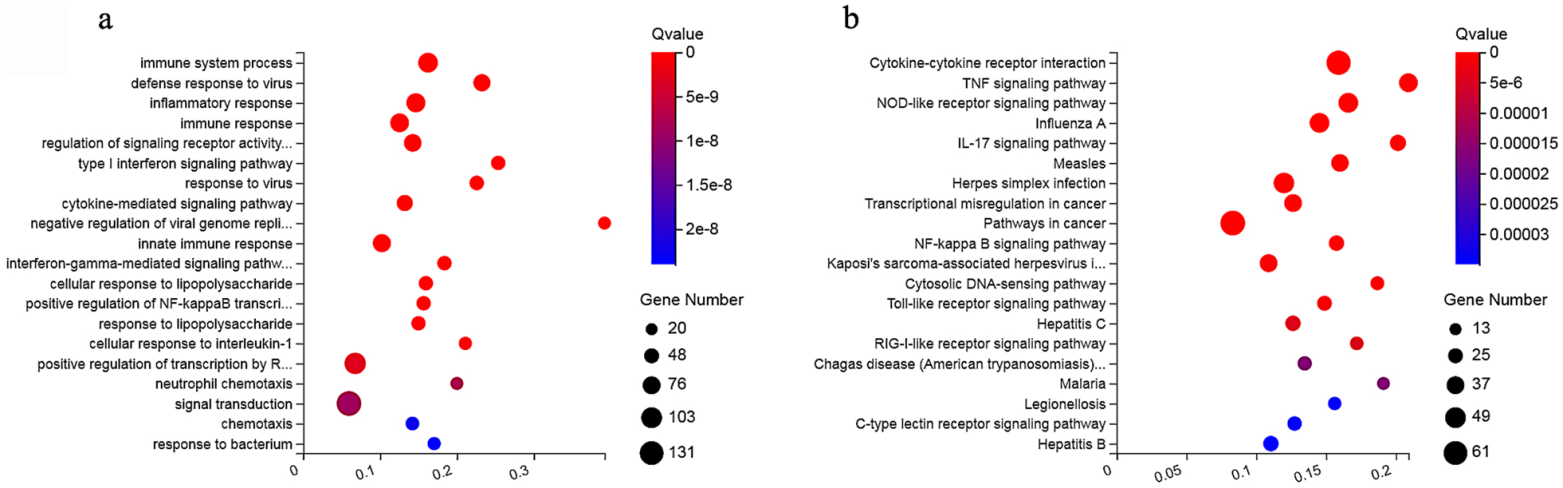
GO enrichment and KEGG pathway analysis of the co-expressed DEmRNAs in HFF cells after *T. gondii* infection. Scatterplots show **a** the 20 most-enriched GO terms in the biological process of DEmRNAs and **b** the top 20 pathways of DElncRNAs. The *x*-axis denotes the pathway enrichment. The *y*-axis represents the names of the significantly enriched GO terms and pathways. The *P*-values are indicated by variations from blue to red, with darker blue denoting more significant difference

The top 20 most-enriched biological processes are shown in Fig. 8a. The biological processes of DEmRNAs were mainly involved in immune-related GO terms, such as the immune system process (e.g., *IFI30*, *TRIM13*, and *DHX36*), defense response to virus (e.g., *TRIM22*, *IFIT5*, and *IFI6*), and inflammatory response (e.g., *NLRP3*, *IFI16*, and *IL-6*). The top 20 most-enriched pathways of DEmRNAs are shown in Fig. 8b. Most DEmRNAs were involved in immune-related pathways such as cytokine-cytokine receptor interaction (e.g., *CSF1*, *CXCL1*, and *IL1A*), TNF signaling (e.g., *FAS*, *IRF1*, and *CXCL3*), and NOD-like receptor signaling (e.g., *GBP4*, *NAMPT* and *BIRC2*).

### Competing endogenous RNA (ceRNA) networks of lncRNAs-mRNAs

**Fig. 9:**
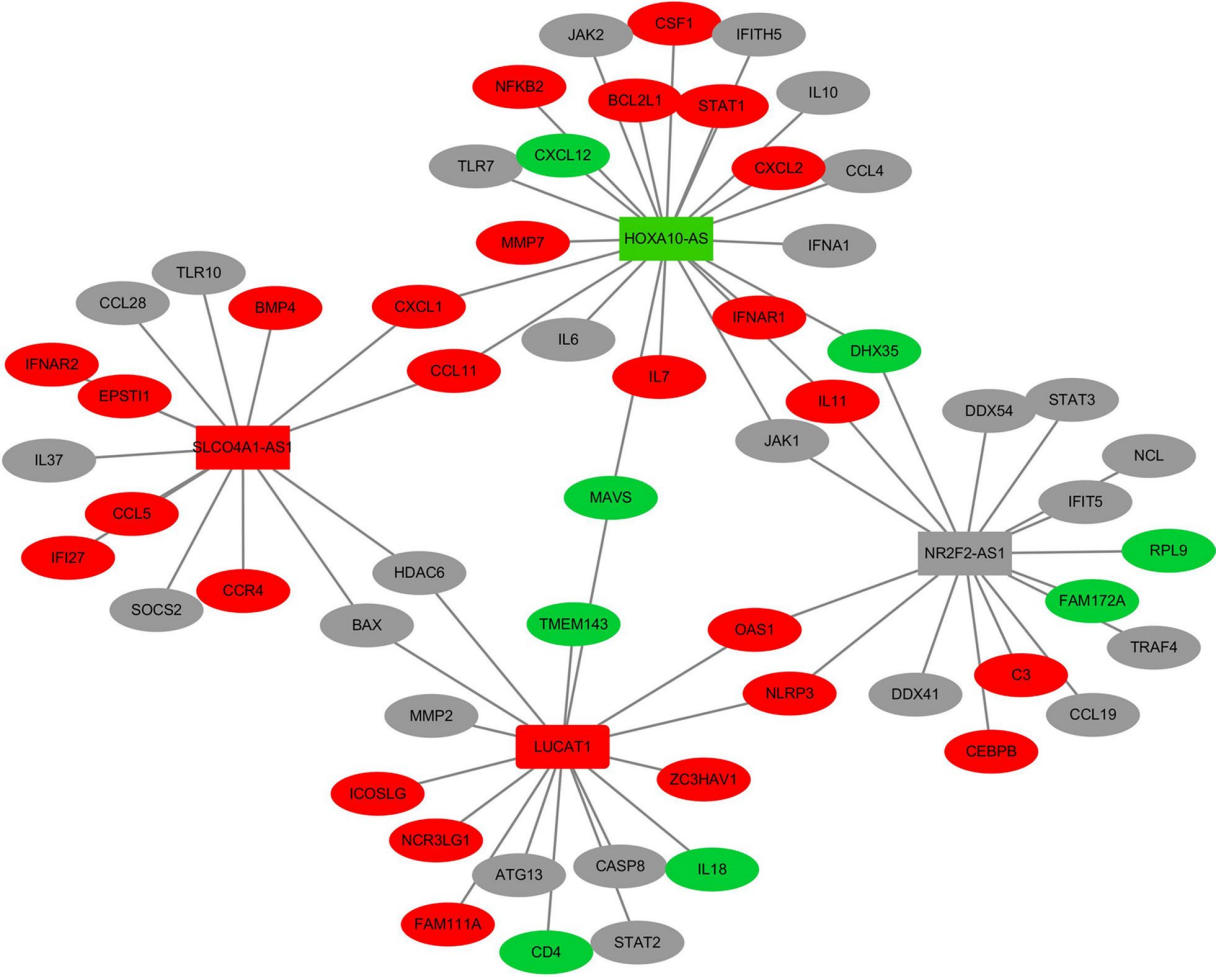
The competing endogenous RNA (ceRNA) networks of lncRNAs and mRNAs. Red and green represent upregulated and downregulated genes, respectively. Gray represents both upregulated and downregulated genes at different times post-infection

To reveal the correlations between DElncRNAs and DEmRNAs, the ceRNA network was constructed using Cytoscape v3.6.0 (2015), based on the potential target relationship between DElncRNAs and DEmRNAs. One lncRNA was associated with one or more mRNAs (Fig. 9).

As shown in Fig. 9, lncRNAs can interact with innate immune-related mRNAs and apoptotic-related mRNAs; for example, *HOXA10-AS* can interact with *MAVS*, *STAT1*, *IL10* and *BCL2L1*. However, the underlying regulatory mechanisms of these DEmRNAs and DElncRNAs need to be elucidated in further studies.

## Discussion

The progressive proliferation of *T. gondii* tachyzoites in the host tissue causes acute infection, disrupting physiological homeostasis and resulting in clinical manifestations such as fever and encephalitis [31]. Understanding how the expression of cellular lncRNAs and mRNAs is altered during *T. gondii* infection can provide new insight into the mechanisms underlying the early stages of the interaction between *T. gondii* and its host. In this study, a total of 7722 DElncRNAs and 16,446 DEmRNAs were identified, and DElncRNAs were mainly involved in immuno-inflammation and metabolism. These results suggest that lnRNAs play a significant role in the pathogenesis of *T. gondii* infection.

Previous studies using transcriptome sequencing detected many DEGs in *T. gondii*-infected animal models [31–33]. These DEGs were mainly involved in immune regulation. The host implements a variety of measures to counter *T. gondii* infection. For example, IFN-inducible GTPase families, including *GBP1*, *GBP2*, *GBP3*, *GBP5*, and *GBP7*, play roles in controlling *T. gondii* infection by regulating IFN-γ-mediated Irgb6-dependent cell-mediated immunity [34]. In the present study, we found that several genes associated with IFN-γ signaling were overexpressed during *T. gondii* infection, including *GBP1*, *GBP2*, *GBP3*, and *GBP5*. We also found that several genes (e.g., *TLR3*, *STAT1*, *DHX58*, *IRF7*, and *Myd88*) involved in the Toll-like receptor signaling pathway, NOD-like receptor signaling pathway, and RIG-I-like receptor signaling pathway were upregulated during *T. gondii* infection (Fig. 6). In addition, inflammation-related genes including *NLRP3*, *IFI16*, *IL6*, *IL1A*, *CXCL2*, and *CCL6* were upregulated, further confirming that infection by *T. gondii* induces a robust immune response to limit infection.

Recent studies have revealed that pathogen invasion can alter the host lnRNA expression, which seems to help pathogens evade the host immune response [35, 36] or enhance the host innate immune response [37]. In the present study, GO and KEGG analysis revealed that many of the DElncRNAs play a role in immune regulation (Fig. 4). *Toxoplasma gondii* can inactivate human plasmacytoid dendritic cells by functional mimicry of *IL-10* [38], and lncRNA *TMC3-AS1* can negatively regulate the expression of *IL-10* [39]. In the present study, lncRNA *TMC3-AS1* was downregulated. The deletion of lncRNA *LUCAT1* in myeloid cells increases the expression of type I interferon-stimulated genes in response to LPS, and overexpression of *LUCAT1* reduces inducible ISG response via their interaction with *STAT1* in the nucleus [40]. In the present study, the expression of *LUCAT1* was upregulated. Previous studies showed that *MAVS* plays a role in inducing an innate immune response and many viruses employ various strategies to suppress its activity [41–43]. In the present study, *T. gondii* downregulated the expression of *MAVS* in infected cells. These findings indicate that *T. gondii* infection can manipulate the host’s immune response by altering the expression of host lncRNAs (e.g., *LUCAT1* and *MAVS*); however, the exact functions of these lncRNAs during *T. gondii* infection remain to be elucidated.

Latent *T. gondii* infection has been associated with various psychiatric disorders in humans [44, 45]. However, the mechanism underlying this connection remains a subject of further investigation [12]. In the present study, *T. gondii* infection increased the expression of genes associated with Alzheimer's disease, Parkinson's disease, and Toxoplasmosis, and genes that play a role in the neurotransmitter release cycle and transmission across chemical synapses. Alterations in solute carrier family genes, such as *SLC2A6*, *SLC1A5*, and *SLC22A4*, have been implicated in the impairment of the synaptic transmission, which seems relevant to the pathology of schizophrenia [46]. Other studies have revealed that altered expression of *DRD1*, which was downregulated in the present study, may contribute to the pathophysiology of schizophrenia and affective disorders [47].

Recent studies have enriched our understanding of the metabolism of both *T. gondii* and its host cells [14, 48, 49]. In the present study, we have shown that *T. gondii* infection changes the host cell’s metabolism by altering the expression of mRNAs and lncRNAs involved in carbon metabolism, metabolic pathways, fatty acid degradation, biosynthesis of secondary metabolites, and pyruvate metabolism (Figs. 4, 6). The tricarboxylic acid (TCA) cycle is essential for *T. gondii* growth [48]. In the absence of glucose, *T. gondii* can utilize the gluconeogenic enzyme fructose bisphosphatase 2 to provide carbon for gluconeogenesis [49, 50]. In the present study, the downregulated lnRNAs were mainly involved in metabolism-related pathways (Additional file 3: Fig. S3), indicating that lncRNAs play a role in regulating host metabolism during *T. gondii* infection.

## Conclusions

Our data revealed significant changes in lncRNA and mRNA expression in HFF cells following *T. gondii* infection. The identified DElncRNAs and DEmRNAs were mainly involved in metabolism, signal transduction, and immune responses. Further investigation of these DElncRNAs and DEmRNAs is warranted to reveal the exact role of these transcripts in the pathophysiology of *T. gondii* infection.

## Supplementary Information

**Additional file 1: Figure S1.** Principal component analysis (PCA) of all identified lncRNAs (**a**) and mRNAs (**b**).

**Additional file 2: Figure S2.** Volcano plots showing the differentially expressed lncRNAs at 1.5, 3, 6, 9, 12, 24, 36, and 48 hpi, respectively (**a-h**). The negative log_10_-transformed *P*-values (*y*-axis) are plotted against the average log_2_ fold changes in expression (*x*-axis). Data points representing lncRNAs that were not differentially expressed are shown in black. Transcripts that are differentially expressed with an absolute |log_2_ fold change (FC)| more than or less than 1 are shown as red (upregulated) and green (downregulated) dots.

**Additional file 3: Figure S3.** Volcano plots showing the differentially expressed mRNAs at 1.5, 3, 6, 9, 12, 24, 36, and 48 hpi, respectively (**a–h**). The negative log_10_-transformed *P*-values (*y*-axis) are plotted against the average log_2_ fold changes in expression (*x*-axis). Data points representing mRNAs that were not differentially expressed are shown in black. Transcripts that are differentially expressed with an absolute |log_2_ fold change (FC)| more than or less than 1 are shown as red (upregulated) and green (downregulated) dots.

**Additional file 4: Table S1.** List of all differentially expressed messenger RNAs (DEmRNAs).

**Additional file 5: Table S2.** List of all differentially expressed long non-coding RNAs (DElncRNAs).

**Additional file 6: Table S3.** List of co-expressed differentially expressed messenger RNAs (DEmRNAs).

**Additional file 7: Table S4.** List of co-expressed differentially expressed long non-coding RNA (DElncRNAs).

**Additional file 8: Figure S4.** KEGG pathway analysis of the target genes of the upregulated DElncRNAs at different time points after infection of HFF cells by *T. gondii*. (**a**–**h**) Scatterplots show the top 20 pathways at 1.5, 3, 6, 9, 12, 24, 36, and 48 hpi, respectively. The *x*-axis denotes the pathway enrichment. The *y*-axis shows the names of the significantly enriched pathways. The *P*-values are indicated by variations from blue to red, with darker blue indicating more significant difference.

**Additional file 9: Figure S5.** KEGG pathway analysis of the target genes of the downregulated DElncRNAs in HFF cells at different time points after *T. gondii* infection. (**a**–**h**) Scatterplots show the top 20 pathways at 1.5, 3, 6, 9, 12, 24, 36, and 48 hpi, respectively. The *x*-axis denotes the pathway enrichment. The *y*-axis shows the names of the significantly enriched pathways. The *P*-values are indicated by variations from blue to red, with darker blue indicating more significant difference.

**Additional file 10: Figure S6.** KEGG pathway analysis of the upregulated mRNAs in HFF cells at different time points after *T. gondii* infection. (**a**–**h**) Scatterplots show the top 20 pathways at 1.5, 3, 6, 9, 12, 24, 36, and 48 hpi, respectively. The *x*-axis denotes the pathway enrichment. The *y*-axis shows the names of the significantly enriched pathways. The *P*-values are indicated by variations from blue to red, with darker blue indicating more significant difference.

**Additional file 11: Figure S7.** KEGG pathway analysis of the downregulated DEmRNAs in HFF cells at different time points after *T. gondii* infection. (**a**–**h**) Scatterplots show the top 20 pathways at 1.5, 3, 6, 9, 12, 24, 36, and 48 hpi, respectively. The *x*-axis denotes the pathway enrichment. The *y*-axis shows the names of the significantly enriched pathways. The *P*-values are indicated by variations from blue to red, with darker blue indicating more significant difference.

## Data Availability

The datasets supporting the findings of this article are included within the article and its additional files. The original data were deposited in the NCBI repository under accession number is PRJNA721229.

## Abbreviations

lncRNAs: Long non-coding RNAs
HFF: Human foreskin fibroblast
DElncRNAs: Differentially expressed lncRNAs
DEmRNAs: Differentially expressed mRNAs
hpi: Hours post-infection
GO: Gene Ontology
KEGG: Kyoto Encyclopedia of Genes and Genomes
PV: Parasitophorous vacuole
ATCC: American Type Culture Collection
DMEM: Dulbecco’s modified Eagle medium
FBS: Fetal bovine serum
MOI: Multiplicity of infection
qRT-PCR: Quantitative real-time PCR
*GAPDH*: Glyceraldehyde-3-phosphate dehydrogenase
*IL-11*: Interleukin-11
*IL-32*: Interleukin-32
*CCL2*: C–C motif chemokine ligand 2
HOXA10: Homeobox A10
*LUCAT1*: Lung cancer-associated transcript 1
*CLMAT3*: Colorectal liver metastasis-associated transcript 3
*TARID*: Transcription factor 21 antisense RNA-inducing promoter demethylation
*CARMN*: Cardiac mesoderm enhancer-associated non-coding RNA
*CFLAR-AS1*: Caspase 8 and Fas associated via death domain like apoptosis regulator antisense RNA 1
*CEBPB-AS1*: CCAAT enhancer binding protein beta antisense RNA 1
*C10orf55*: Chromosome 10 putative open reading frame 55
*CHKB-DT*: Choline kinase beta divergent transcript
*IL6*: Interleukin-6
TMEM161B-AS1: transmembrane protein 161B antisense RNA 1
*CXCL10*: C–X–C motif chemokine ligand 10
MAP3K8: mitogen-activated protein kinase kinase kinase 8
*CSF2*: Colony-stimulating factor 2
IL-17: Interleukin-17
*NFKB1*: Nuclear factor kappa B subunit 1
*FOSL1*: FOS-like 1
*GBP1*: Guanylate binding protein 1
*GBP2*: Guanylate binding protein 2
*IFNAR2*: Interferon alpha and beta receptor subunit 2
*EGOT*: Eosinophil granule ontogeny transcript
*LYRM4-AS1*: LYR motif-containing 4 antisense RNA 1
*SLCO4A1-AS1*: Solute carrier organic anion transporter family member 4A1 antisense RNA 1
*MSC-AS1*: Musculin antisense RNA 1
*GNG12-AS1*: G protein subunit gamma 12
*PLCE1-AS1*: Phospholipase C epsilon 1 antisense RNA 1
*ZNF667-AS1*: Zinc finger protein 667 antisense RNA 1
*ITGB1-DT*: Integrin subunit beta 1 divergent transcript
*IFI30*: Interferon gamma lysosomal thiol reductase
*TRIM13*: Tripartite motif-containing 13
*DHX36*: DEAH-box helicase 36
*TRIM22*: Tripartite motif-containing 22
*IFIT5*: Interferon-induced protein with tetratricopeptide repeats 5
*IFI6*: Interferon-induced protein with tetratricopeptide repeats 6
*NLRP3*: NOD-like receptor family pyrin domain-containing 3
*IFI16*: Interferon-induced protein with tetratricopeptide repeats 16
*CSF1*: Colony-stimulating factor 1
*CXCL1*: C–X–C motif chemokine ligand 1
*IL1A*: Interleukin 1 alpha
*FAS*: Fas cell surface death receptor
*IRF1*: Interferon regulatory factor 1
*CXCL3*: C–X–C motif chemokine ligand 3
*GBP4*: Guanylate-binding protein 4
*NAMPT*: Nicotinamide phosphoribosyltransferase
*BIRC2*: Baculoviral IAP repeat-containing 2
IFN-γ: Interferon gamma
*GBP3*: Guanylate-binding protein 3
*GBP5*: Guanylate-binding protein 5
*GBP7*: Guanylate-binding protein 7
*TLR3*: Toll-like receptor 3
*STAT1*: Signal transducer and activator of transcription 1
*DHX58*: DExH-box helicase 58
*IRF7*: Interferon regulatory factor 7
*Myd88*: Myeloid differentiation primary response gene 88
*CXCL2*: C–X–C motif chemokine ligand 2
*CCL6*: Chemokine (C–C motif) ligand 6
*TMC3-AS1*: Transmembrane channel-like 3 antisense RNA 1
LPS: Lipopolysaccharides
ISG: Interferon-stimulated gene
*MAVS*: Mitochondrial antiviral signaling protein
SLC2A6: Solute carrier family 2 member 6
*SLC1A5*: Solute carrier family 2 member 5
*SLC22A4*: Solute carrier family 22 member 4
DRD1: Dopamine receptor D1
TCA: Tricarboxylic acid
ceRNA: Competing endogenous RNA
