# Supplementary figures and images for: Temporal transcriptomic changes in long non-coding RNAs and messenger RNAs involved in the host immune and metabolic response during Toxoplasma gondii lytic cycle
Source: Parasit Vectors. 2022 Jan 10;15:22. doi: 10.1186/s13071-021-05140-3 (PMC8750853; doi:10.1186/s13071-021-05140-3)

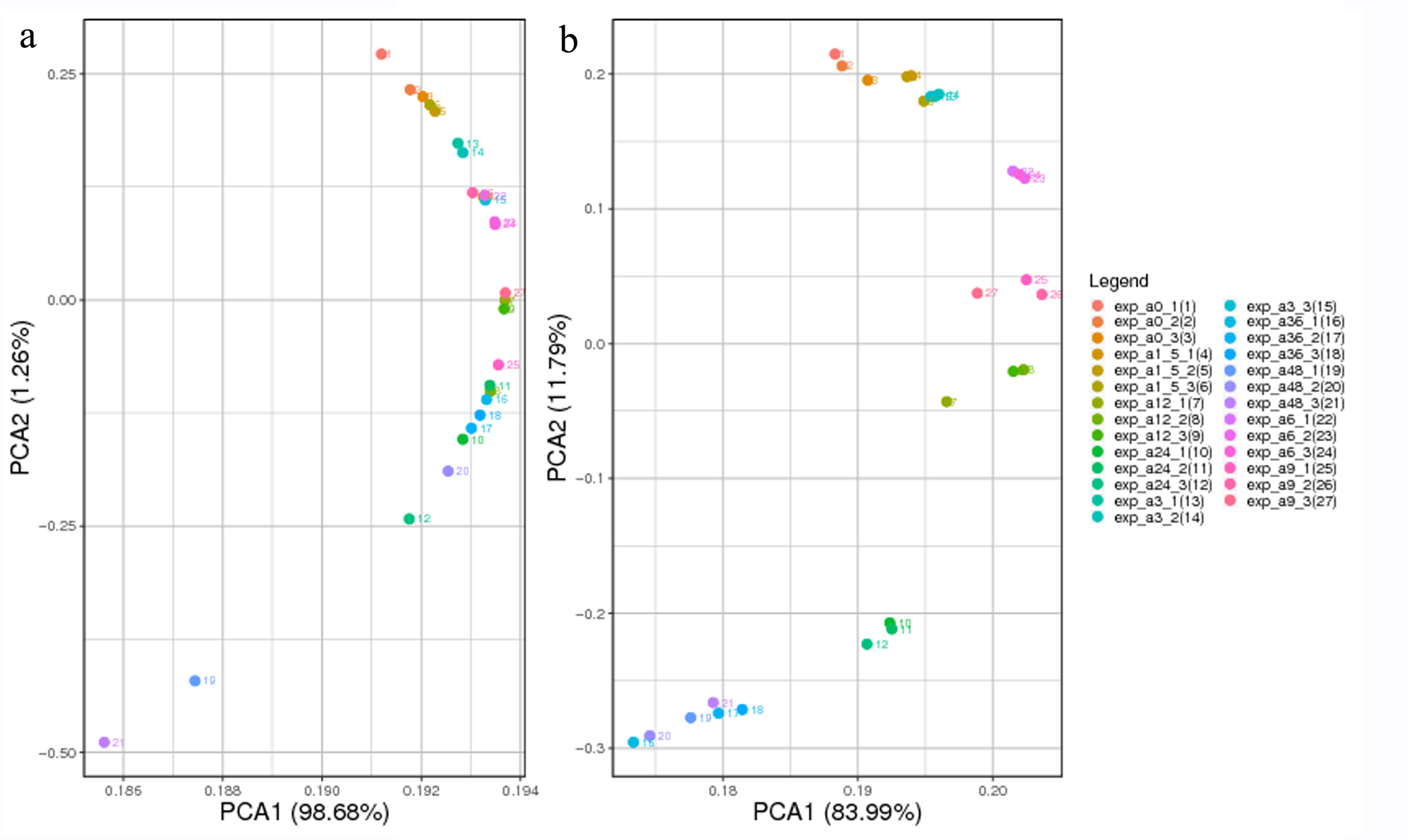

Supplement: Supplementary file 1 — Additional file 1: Figure S1. Principal component analysis (PCA) of all identified lncRNAs (a) and mRNAs (b). [file 13071_2021_5140_MOESM1_ESM.tif]

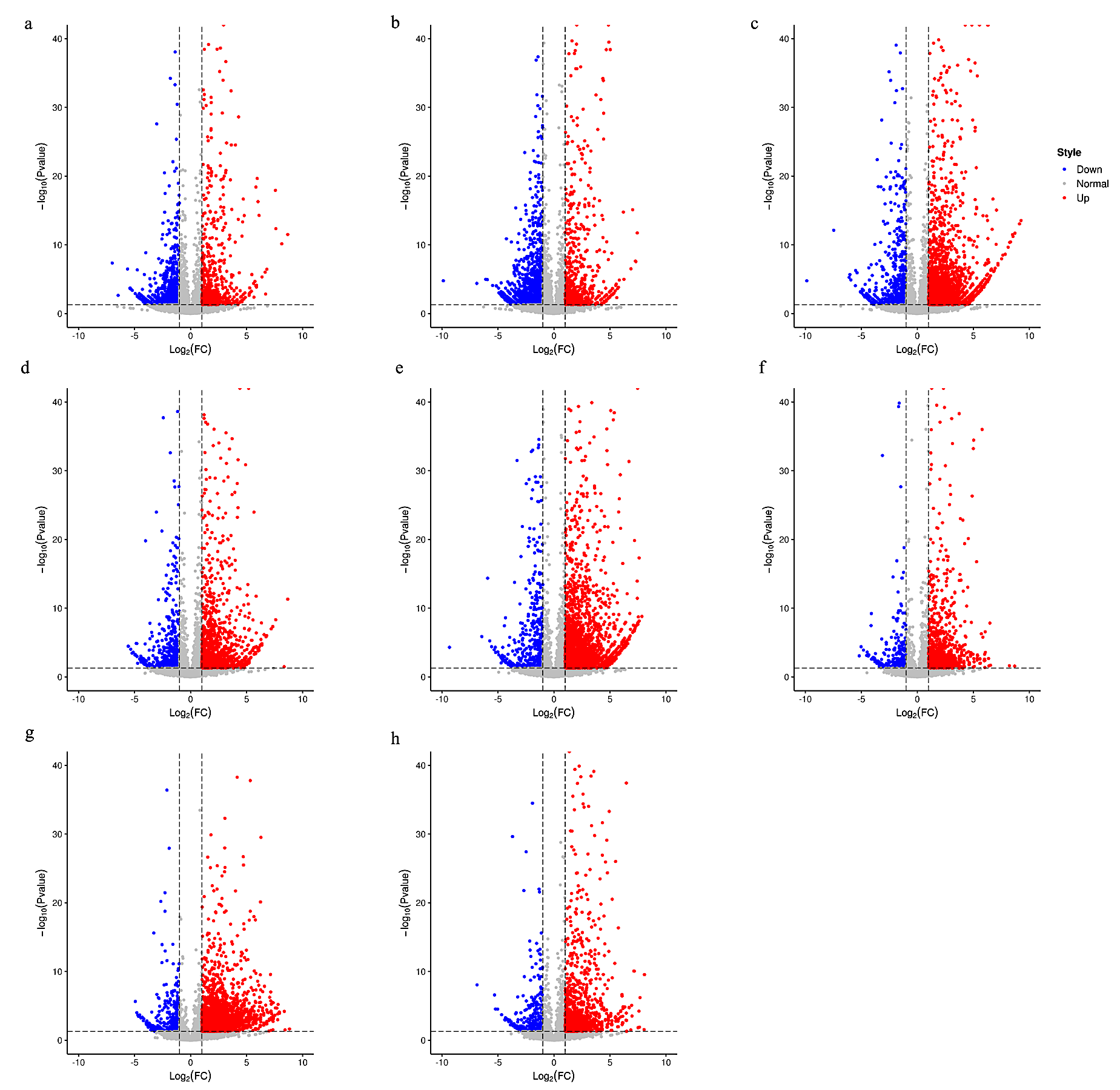

Supplement: Supplementary file 2 — Additional file 2: Figure S2. Volcano plots showing the differentially expressed lncRNAs at 1.5, 3, 6, 9, 12, 24, 36, and 48 hpi, respectively (a-h). The negative log10-transformed P-values (y-axis) are plotted against the average log2 fold changes in expression (x-axis). Data points representing lncRNAs that were not differentially expressed are shown in black. Transcripts that are differentially expressed with an absolute |log2 fold change (FC)| more than or less than 1 are shown as red (upregulated) and green (downregulated) dots. [file 13071_2021_5140_MOESM2_ESM.tif]

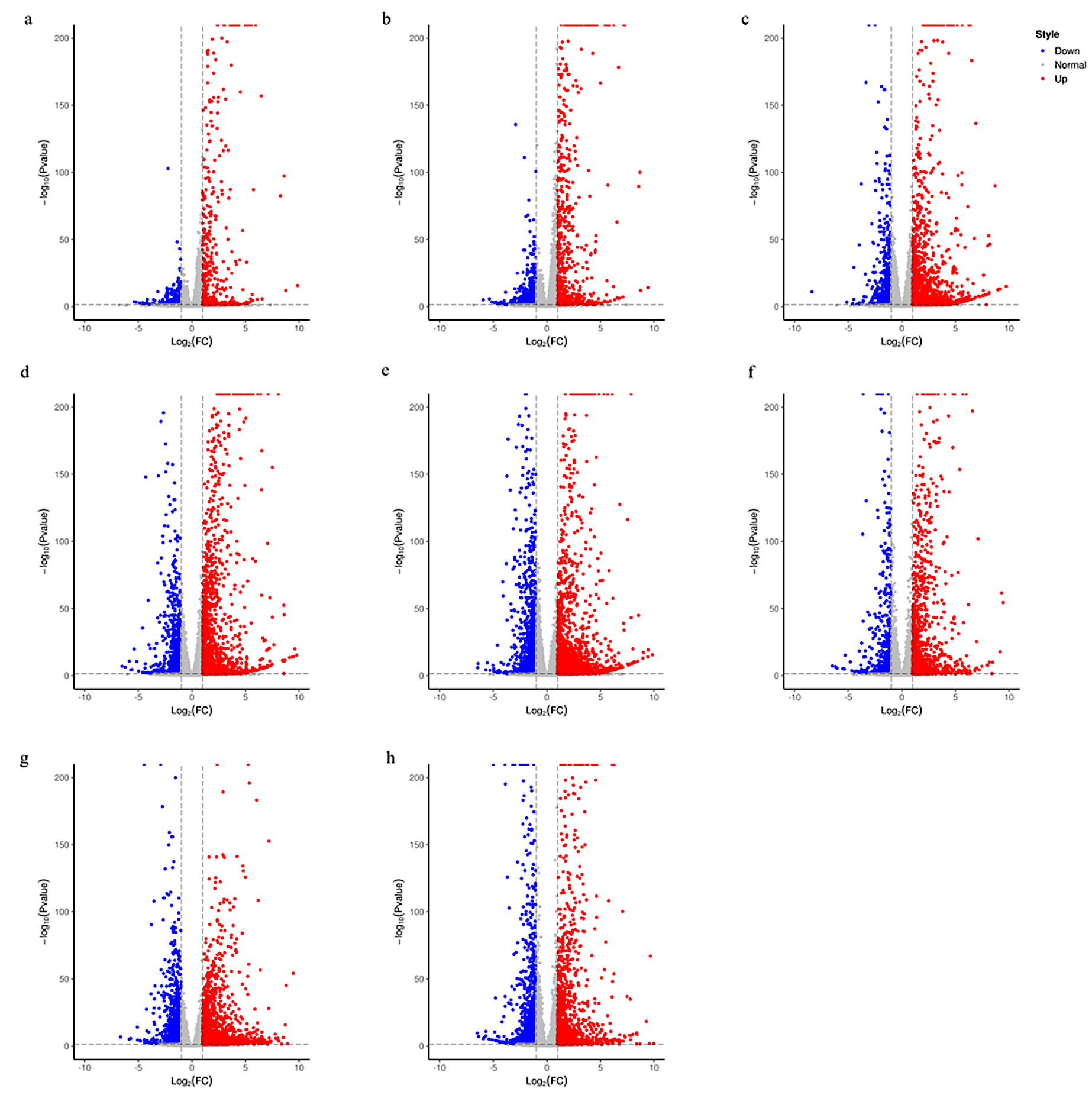

Supplement: Supplementary file 3 — Additional file 3: Figure S3. Volcano plots showing the differentially expressed mRNAs at 1.5, 3, 6, 9, 12, 24, 36, and 48 hpi, respectively (a–h). The negative log10-transformed P-values (y-axis) are plotted against the average log2 fold changes in expression (x-axis). Data points representing mRNAs that were not differentially expressed are shown in black. Transcripts that are differentially expressed with an absolute |log2 fold change (FC)| more than or less than 1 are shown as red (upregulated) and green (downregulated) dots. [file 13071_2021_5140_MOESM3_ESM.tif]

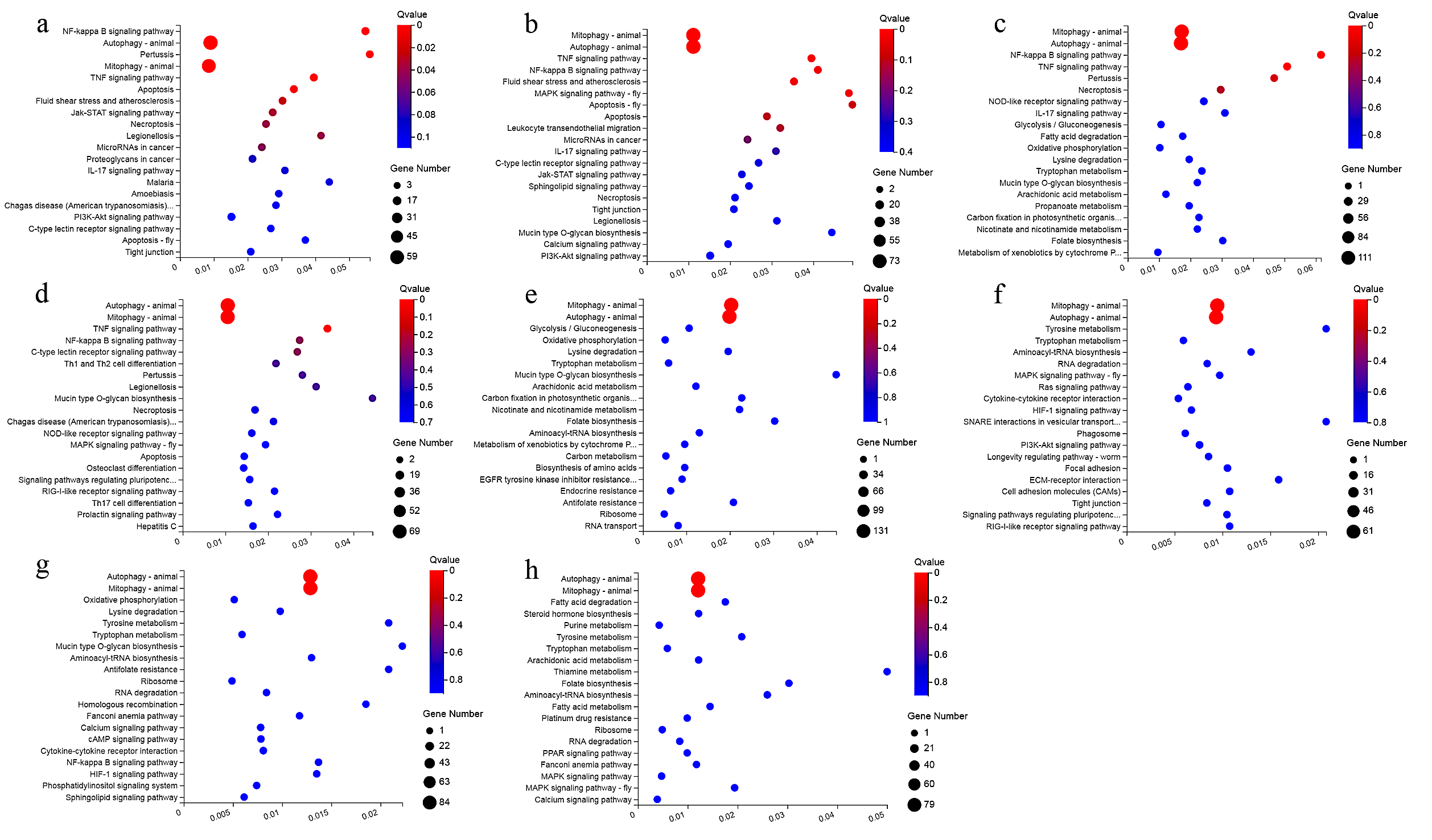

Supplement: Supplementary file 8 — Additional file 8: Figure S4. KEGG pathway analysis of the target genes of the upregulated DElncRNAs at different time points after infection of HFF cells by T. gondii. (a–h) Scatterplots show the top 20 pathways at 1.5, 3, 6, 9, 12, 24, 36, and 48 hpi, respectively. The x-axis denotes the pathway enrichment. The y-axis shows the names of the significantly enriched pathways. The P-values are indicated by variations from blue to red, with darker blue indicating more significant difference. [file 13071_2021_5140_MOESM8_ESM.tif]

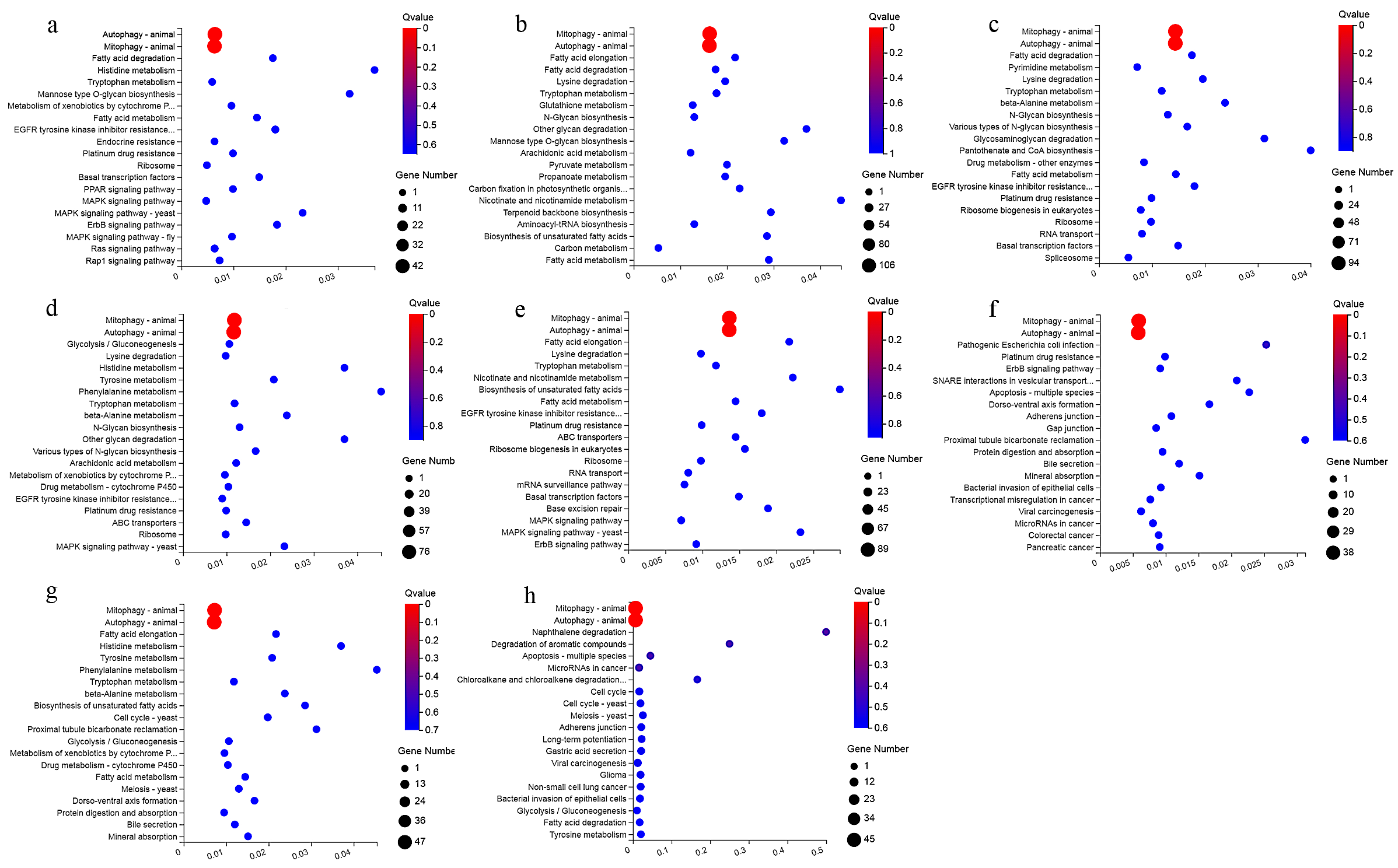

Supplement: Supplementary file 9 — Additional file 9: Figure S5. KEGG pathway analysis of the target genes of the downregulated DElncRNAs in HFF cells at different time points after T. gondii infection. (a–h) Scatterplots show the top 20 pathways at 1.5, 3, 6, 9, 12, 24, 36, and 48 hpi, respectively. The x-axis denotes the pathway enrichment. The y-axis shows the names of the significantly enriched pathways. The P-values are indicated by variations from blue to red, with darker blue indicating more significant difference. [file 13071_2021_5140_MOESM9_ESM.tif]

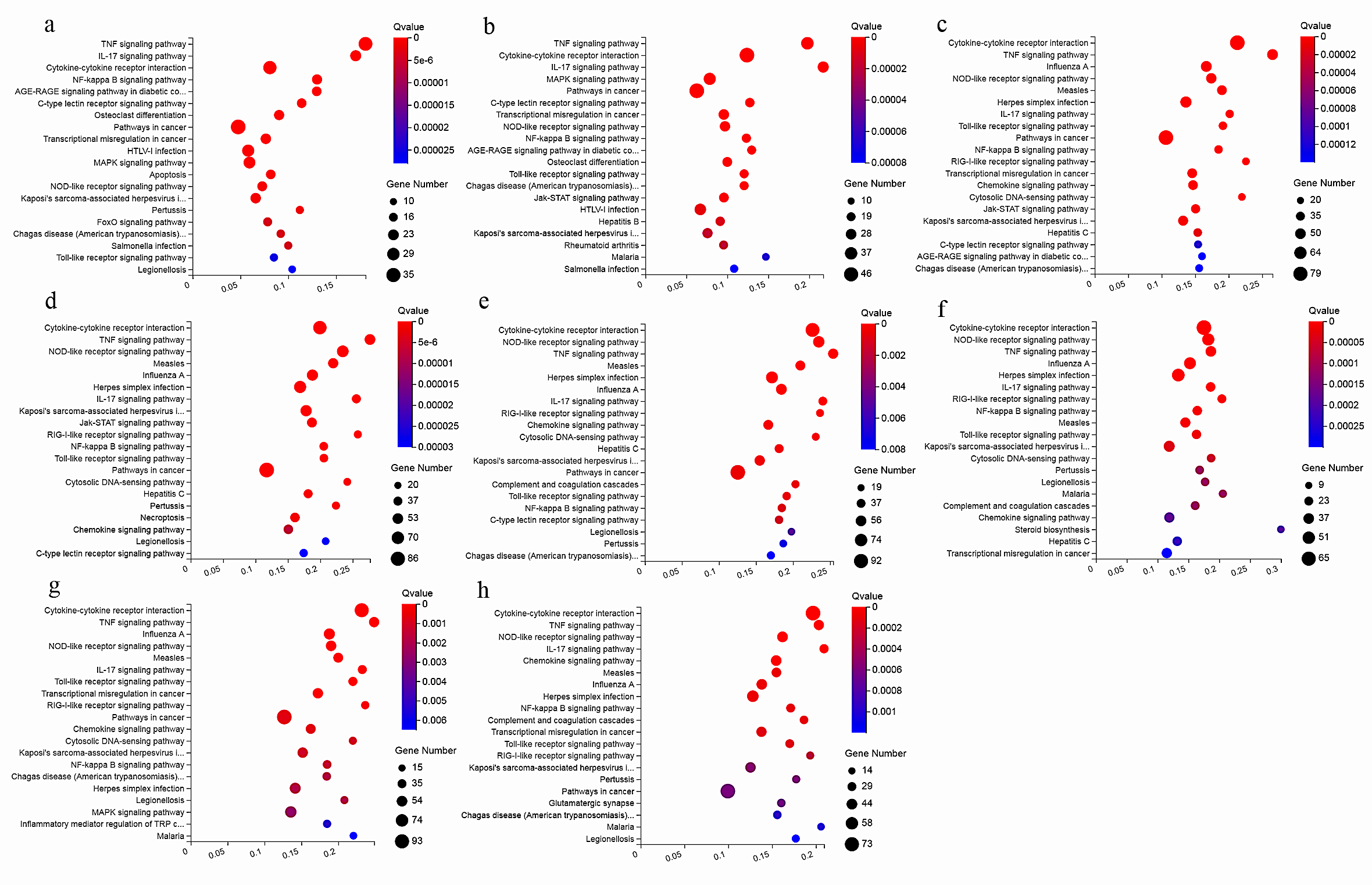

Supplement: Supplementary file 10 — Additional file 10: Figure S6. KEGG pathway analysis of the upregulated mRNAs in HFF cells at different time points after T. gondii infection. (a–h) Scatterplots show the top 20 pathways at 1.5, 3, 6, 9, 12, 24, 36, and 48 hpi, respectively. The x-axis denotes the pathway enrichment. The y-axis shows the names of the significantly enriched pathways. The P-values are indicated by variations from blue to red, with darker blue indicating more significant difference. [file 13071_2021_5140_MOESM10_ESM.tif]

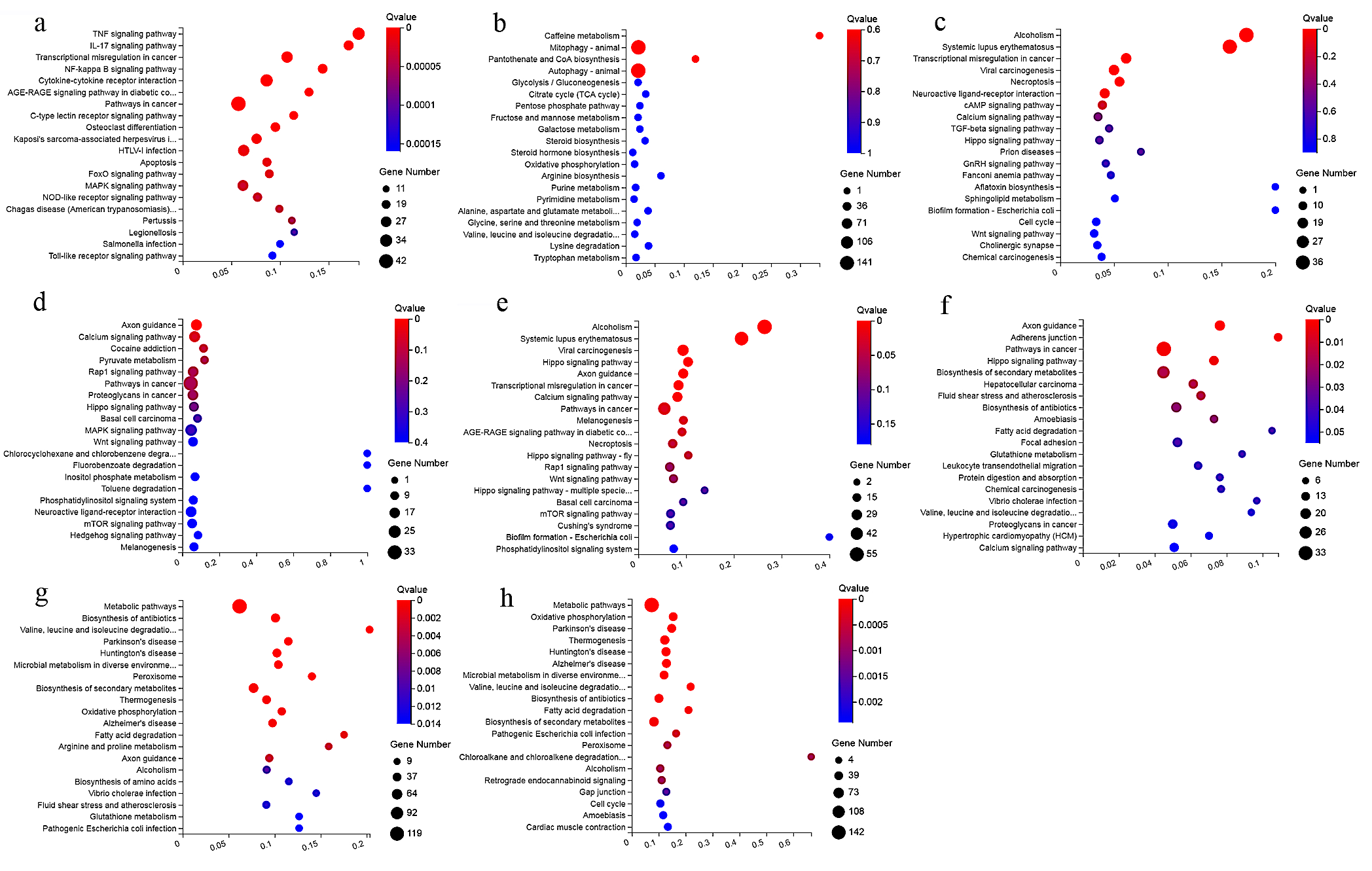

Supplement: Supplementary file 11 — Additional file 11: Figure S7. KEGG pathway analysis of the downregulated DEmRNAs in HFF cells at different time points after T. gondii infection. (a–h) Scatterplots show the top 20 pathways at 1.5, 3, 6, 9, 12, 24, 36, and 48 hpi, respectively. The x-axis denotes the pathway enrichment. The y-axis shows the names of the significantly enriched pathways. The P-values are indicated by variations from blue to red, with darker blue indicating more significant difference. [file 13071_2021_5140_MOESM11_ESM.tif]
